# A New Pathology in Epilepsy

**Published:** 1898-10

**Authors:** 


### A New Pathology in Epilepsy.

Epilepsy, one of the oldest diseases of the nervous system of which we have knowledge, is, as regards its etiology, and particularly its pathology, practically but little known. The names given it by the Father of Medicine, "sacred malady" and "malady of children," show in what light it was regarded by the ancients. For centuries the most ridiculous and incredible theories were advanced by the best men of their time with truly commendable zeal; but real progress cannot be said to have begun previous to the statements of reflex irritation advocated by Marshall Hall. Since then decided advances have been made along many lines, due especially to the magnificent work of Hughlins-Jackson, but even to this day authorities differ greatly as to causation in a vast majority of those afflicted. Age, sex, heredity, insanity, organic brain disease, trauma, syphilis, chronic alcoholism of parents, must all be regarded as important considerations. Many curious facts in respect to etiology have been reported of late years; errors of refraction, especially astigmatism, are of common occurrence; compression of the carotids producing cerebral anemia; bradycardia; diabetes; the diminution of the toxicity of the urine previous to an attack; pressure upon the undescended testicle; all in a considerable number of instances by a number of observers. The role of trauma in the causation of epilepsy is too well grounded to be easily shaken, but undoubtedly too many cases are classed under this head. Almost every individual, whether epileptic or not, can give a history either known to himself or narrated by his parents or friends, of a fall upon the head during childhood or at some later period, and yet but a small proportion of the population suffer from epilepsy. Probably only a small ratio of epileptics, not over two per cent, could rightfully attribute their affliction to this cause. There remains, then, the major portion of the sufferers, in whom the etiology of their affliction and certainly pathology, is obscure, that must be classed under the term—for want of a better—idiopathic epilepsy. For the clearing up of obscure points of this disease, too much attention has doubtless been directed to the nervous system, as is the case of insanity, with a passing over with but trifling consideration other pathologic conditions existing in the body.

For the study of epilepsy, both the clinical manifestations during life and the pathological findings after death, there is probably no better place in the country than the Ohio State Hospital for Epileptics at Gallipolis. There are usually from six to seven hundred inmates, twenty or thirty of whom come to the autopsy table during the year. Lately a well-appointed laboratory has been fitted up, and the research work placed under the able direction of Dr. A. Ohlmacher. The latter, in his researches into epilepsy, determined to examine not only the central nervous system, but all other parts of the body as well, with the hope that some constant pathologic lesion might be found upon which statistics of some value could be compiled. His efforts have been crowned with considerable success, as demonstrated by his report in the *Columbus Medical Journal* of July 19, "The Persistent Thymus and Other Morbid Anatomic Peculiarities in Certain Cases of Epilepsy." His studies comprise eighteen cases, in eight of which more or less constant pathologic features were presented. All the victims were in the young adult period of life. Six of the eight were undoubtedly mrand mal, therefore "idiopathic." A curious fact is that three of the eight died suddenly. One other committed suicide and one died of exhaustion after an attack of mania. Four of the eight had attacks of mania at more or less regular intervals. The summary of his findings can be perhaps best stated in his own words: "Persistent and enlarged thymus gland; a pronounced enlargement of the intestinal and splenic lymph-follicles; a more or less pronounced hypertrophy of the lymphatic glands, and the lymph-adenoid follicles of the tongue, larynx, trachea, esophagus, tonsils, and even of the stomach; a narrowing of the arteries; an abundant development of fat; and certain osseous changes indicative of old rickets. Not all the abnormalities comprehended in this summary were present in a single case, though the persistent thymus, with one or several features added, was constant." Ohlmacher, in conclusion, calls attention to enlarged thymus occurring in two other conditions, namely, "laryngismus stridulous" and "sudden death in adults with no assignable lesion," and lays special stress upon the sudden death of his three epileptics, in all of whom it might again be remarked that the thymus was enlarged. He regards the "lymphatic constitution" occurring in these three affections and the sudden dissolution that appears so commonly as more than a mere coincidence. It is to be hoped that other institutions will direct their attention to the findings of Ohlmacher, so that the great pedestal for the successful treatment of any disease, a morbid anatomy, may in this most obscure of affections be founded upon a firm basis.—Editorial, *Journal of the American Medical Association.*

